# Protection against SARS-CoV-2 transmission by a parenteral prime—Intranasal boost vaccine strategy

**DOI:** 10.1016/j.ebiom.2022.104248

**Published:** 2022-09-07

**Authors:** Dennis Christensen, Charlotta Polacek, Daniel J. Sheward, Leo Hanke, Ainhoa Moliner-Morro, Gerald McInerney, Ben Murrell, Katrine Top Hartmann, Henrik Elvang Jensen, Gregers Jungersen, Kristin Illigen, Louise Krag Isling, Rune Fledelius Jensen, Julia Sid Hansen, Ida Rosenkrands, Carlota Fernandez-Antunez, Santseharay Ramirez, Frank Follmann, Jens Bukh, Gabriel Kristian Pedersen

**Affiliations:** a Center for Vaccine Research, Statens Serum Institut, Copenhagen, Denmark; b Virus Research & Development Laboratory, Department of Virology and Microbiological Special diagnostics, Statens Serum Institut, Copenhagen, Denmark; c Department of Microbiology, Tumor and Cell Biology, Karolinska Institutet, Stockholm, Sweden; d Department of Veterinary and Animal Sciences, University of Copenhagen, Copenhagen Denmark; e Copenhagen Hepatitis C Program (CO-HEP), Department of Infectious Diseases, Copenhagen University Hospital, Hvidovre, and Department of Immunology and Microbiology, Faculty of Health and Medical Sciences, University of Copenhagen, Copenhagen, Denmark

**Keywords:** SARS-CoV-2, CAF®01, Neutralizing antibodies, Intranasal vaccine, Onward transmission

## Abstract

**Background:**

Licensed vaccines against SARS-CoV-2 effectively protect against severe disease, but display incomplete protection against virus transmission. Mucosal vaccines providing immune responses in the upper airways are one strategy to protect against transmission.

**Methods:**

We administered Spike HexaPro trimer formulated in a cationic liposomal adjuvant as a parenteral (subcutaneous – s.c.) prime - intranasal boost regimen to elicit airway mucosal immune responses and evaluated this in a Syrian hamster model of virus transmission.

**Findings:**

Parenteral prime - intranasal boost elicited high-magnitude serum neutralizing antibody responses and IgA responses in the upper respiratory tract. The vaccine strategy protected against virus in the lower airways and lung pathology, but virus could still be detected in the upper airways. Despite this, the parenteral prime - intranasal booster vaccine effectively protected against onward SARS-CoV-2 transmission.

**Interpretation:**

This study suggests that parenteral-prime mucosal boost is an effective strategy for protecting against SARS-CoV-2 infection and highlights that protection against virus transmission may be obtained despite incomplete clearance of virus from the upper respiratory tract. It should be noted that protection against onward transmission was not compared to standard parenteral prime-boost, which should be a focus for future studies.

**Funding:**

This work was primarily supported by the European Union Horizon 2020 research and innovation program under grant agreement no. 101003653.

**Research in context**

**Evidence before this study**

Licensed vaccines against SARS-CoV-2 are highly effective at preventing severe disease, but fail to completely protect against virus transmission. Subunit vaccines incorporating an effective mucosal adjuvant can induce mucosal responses in the upper respiratory tract, which have the potential to block respiratory pathogens at the portal of entry. Preclinical models recapitulating natural infection are needed to study the capacity of vaccines to block virus transmission.

**Added value of this study**

Our study demonstrates that a parenteral prime - mucosal boost vaccine strategy can protect against SARS-CoV-2 infection and pathology in the lower respiratory tract. Furthermore, onward transmission from vaccinated animals was significantly reduced compared to that observed in unvaccinated controls. Our study did not directly assess if parenteral prime - mucosal boost was superior to parenteral only immunization for protecting against onward transmission.

**Implications of all the available evidence**

The study suggests that a parenteral prime - mucosal booster strategy using protein-based subunit vaccines may be an effective means to protect against transmission of SARS-CoV-2 and potentially other respiratory viruses.

Alt-text: Unlabelled box

## Introduction

Licensed Acute Respiratory Syndrome Coronavirus 2 (SARS-CoV-2) vaccines relying on novel technologies, including messenger RNA vaccines,^1^, ^2^, ^3^ have proved highly effective against severe COVID-19. However, a major limitation of these vaccines is their lower effectiveness at protecting against virus transmission than against disease. Parenteral vaccines predominantly induce systemic IgG antibody responses, but respiratory viruses with pandemic potential, including coronaviruses, are mainly transmitted from person to person via respiratory droplets and infect the upper respiratory tract, which is not effectively protected by circulating IgG.^4^^,^^5^ Failure to elicit sterilizing immunity can lead to local viral replication in respiratory tissues and potentially onward transmission, enabling the development and spread of resistant variants. Mucosal vaccination is an established strategy for induction of secretory IgA (sIgA) at the mucosal surfaces that, by blocking virus at the portal of entry, may prevent initial viral replication and thus potentially provide sterilizing immunity. Compared to the monomeric IgG, sIgA is multimeric, providing increased avidity and sIgA thus can be better at neutralizing SARS-CoV-2 than IgG.^6^^,^^7^ Intranasal (i.n.) immunization also elicits local tissue resident (T_RM_) CD4 and CD8 T cell responses in nasal-associated lymphoid tissue (NALT).^8^^,^^9^ For SARS-CoV-1, an i.n. vaccine induced respiratory CD4 T cells recruiting protective CD8 T cells to NALT via an IFN-γ dependent mechanism.^10^ The Th17 cell subset has received particular focus in mucosal immune responses^11^ and Th17-produced IL-17A upregulates polymeric immunoglobulin receptor (pIgR) to promote secretory IgA responses.^12^, ^13^, ^14^ One strategy to facilitate both systemic immunity and mucosal immune responses in the upper airways is by a parenteral prime – i.n. boost regimen.^11^^,^^15^, ^16^, ^17^ We tested this strategy for SARS-CoV-2, using a cationic liposome (CAF®01) adjuvanted spike subunit vaccine. Immunization by parenteral prime – i.n. boost induced anti-spike IgG and SARS-CoV-2 neutralizing antibody responses in serum and elicited IgA responses in the upper respiratory tract. In a transmission model in which vaccinated contacts were co-housed with SARS-CoV-2 infected index hamsters, the parenteral prime-mucosal boost strategy lowered virus titres in the upper airways and protected against onward transmission. Overall, a parenteral prime - i.n. boost vaccine strategy may be an effective means to limit virus spread in the population.

## Methods

### Ethics

Animal studies were conducted in accordance with European Community Directive 2010/63/EU. The experiments have been approved by, and conducted in compliance with, the governmental Animal Experiments Inspectorate under licenses 2017-15-0201-01363 and 2020-15-0201-00554.

### Antigens and adjuvants

Recombinant SARS-CoV-2 prefusion-stabilized spike ectodomain (S-2P^18^ and HexaPro trimer^19^), and the RBD domain (RVQ-VNF) from the Wuhan-Hu-1 strain were produced by transient expression in freestyle 293-F cells, as reported previously.^20^^,^^21^ CAF®01 (250 μg DDA/ 50 μg TDB) in 10mM TRIS buffer with 2.2% glycerol (pH 7.0) was produced as described previously.^22^

### Characterization of formulations

A compatibility study of the spike HexaPro trimer in CAF®01 was performed at room temperature. Formulations were analysed visually for potential flocculation and then characterized for particle size and polydispersity index (PDI) by dynamic light scattering, using the photon correlation spectroscopy technique. The zeta potential was measured by laser-Doppler electrophoresis. For the size measurements, the samples were diluted 10 times, whereas for the zeta potential measurements, the samples were diluted 100 times in milli-Q water. The measurements were performed at 25 °C, using a Zetasizer Nano ZS (Malvern Instruments, Worcestershire, UK) with a 633 nm laser and 173° detection optics. Malvern Zetasizer software v.8.01 was used for analysis.

### Animals

Female C57Bl/6 (C57BL/6JOlaHsd) wild type mice, 7–9 weeks old, were obtained Envigo (The Netherlands). Male Syrian golden hamsters (*Mesocricetus auratus*), nine weeks old, were obtained from Janvier. Both species were housed in the animal facilities at Statens Serum Institut, Denmark during the studies and maintained in rooms with controlled environment (20–23 °C; relative humidity 52 ± 10%; 12/12 h light/dark cycle). The mice were randomly allocated to cages (type III polycarbonate cages (820 cm^2^)) with up to eight mice per cage and the hamsters were housed in polycarbonate cages type IV (1820 cm^2^) with high lid (in total app. 30 cm. high) with up to four animals/cage. A total of 28 mice and 51 hamsters were used for the studies. All animals were offered Aspen bedding and bricks (Tapvei), Sizzelnest (Datesand) and tunnels or houses made of polycarbonate. Besides, mice were offered DesRes paper houses (LBS) while hamsters had twisted paper rolls (“Diamond Twist” Envigo Teclad) hanging in their cage lids. Irradiated sunflower seeds, corn grains and peanuts or bits of carrots were offered all the animals once a week.

Pelleted diet (Envigo Teclad 2916) and tap water was provided ad libitum.

### Immunizations

Mice and hamsters were administered with two immunizations 21 days apart, using 5 μg (mice) or 20 μg (hamsters) of recombinant SARS-CoV-2 prefusion-stabilized spike ectodomain formulated in CAF®01,^22^ as described previously.^23^ The spike S-2P variant was used in initial mouse studies, whilst the HexaPro variant was used in all hamster studies and in mouse studies where indicated. The vaccine was either given as two subcutaneous (s.c.) immunizations (s.c./s.c.) or as s.c. immunization followed by intranasal (i.n.) boost (s.c./i.n.). The s.c. immunizations were given at the base of tail (mice) or in the scruff of the neck (hamsters) at a final volume of 200 μl per immunization. The i.n. immunizations were performed under isofluorane anaesthesia. Mice were given a final volume of 20 μl (10 μl per nostril). Hamsters were placed in supine position and given 50 μl (25 μl per nostril). The number of animals in each group is stated in the figure legends. Power calculations were based on levels of antigen-specific IgA (against another protein antigen) in s.c./i.n. immunized compared to unvaccinated controls in a previous mouse study with effect size (OD_450_) of 0.58 and SD of 0.297. An analysis plan was prepared for each study as part of the study protocol.

### Sampling and organ preparation from mice

Mice were euthanized by CO_2_ (80%)/O_2_ (20%), after anaesthetization with Zoletil-mix (Zolazepam, Tiletamin, Xylazin and Butorphanol). Lungs and spleens were filtered through a 70 μm nylon mesh (BD Biosciences). The cells were washed and re-suspended in cell culture medium (RPMI-1640 supplemented with 2-mercaptoethanol, 1% pyruvate, 1% HEPES, 1% (v/v) premixed penicillin-streptomycin solution (Invitrogen Life Technologies), 1 mM glutamine, and 10% (v/v) fetal calf serum (FCS)). For Meso Scale Discovery (MSD) cytokine profiling, cells were added in 200 μl of cell culture medium into 96-well cell culture plates at a density of 2 × 10^5^ cells/well. Nasal washes were sampled by flushing with 350 µl phosphate buffered saline (PBS) +0.05% bovine serum albumin.

### ELISA for spike-ACE2 binding

ACE2 binding ELISA was performed to assess if spike-ACE2 binding was intact after formulation of spike in CAF®01 liposomes. Maxisorp Plates (Nunc, Denmark) were coated overnight with recombinant human ACE2 (ACE-HM101, Kactus Biosystems) at 2 µg/ml in carbonate buffer pH 9.6. Plates were washed with PBS containing 0.2% Tween 20 and blocked with 2% skimmed-milk powder (SM). After blocking, spike protein alone or spike protein formulated in CAF®01 were added serially diluted in PBS with 1% SM, followed by incubation with hamster-anti-spike protein serum (generated in-house by immunizing hamsters with recombinant spike protein) as primary antibody. HRP-conjugated goat anti-hamster IgG antibody (Invitrogen, AB_2536572) was used as secondary antibody. TMB-PLUS (Kem-En-TEC, Denmark) was used as substrate, and the absorbance was recorded at 450 nm with subtraction of the absorbance value measured at 620 nm.

### ELISA for antibody responses

Maxisorb Plates (Nunc) were coated overnight with 0.05 μg/well SARS-CoV-2 trimer or RBD (4 °C). For antigen-specific IgG, plates were blocked with 2% BSA and serum was added diluted in PBS with 1% BSA. Polyclonal HRP-conjugated secondary rabbit anti-mouse IgG (Thermofisher, RRID:AB_138451) or goat anti-hamster IgG antibody (Invitrogen, AB_2536572), was diluted in PBS with 1% BSA. For antigen-specific IgA, plates were blocked with 2% milk, and serum and nasal washes were added in PBS with 1% milk. Biotin-coupled goat anti-mouse IgA (Southern Biotech, AB_2794374) diluted in 1% milk was used as primary antibody, followed by streptavidin-HRP (BD, AB_2868972) diluted in PBS containing 0.2% Tween 20.

After incubation of secondary antibodies, Spike- or RBD-specific antibodies were detected using TMB substrate as described by the manufacturer (Kem-En-Tec Diagnostics), and the reaction was stopped with H_2_SO_4_. The absorbance was measured at 450 nm with subtraction of the absorbance value measured at 620 nm.

### Cytokine profiling

The Mouse U-plex (IFN-γ, IL-17A, IL-5, IL-13 and IL-10) was performed according to the manufacturer's instructions (Meso Scale Discovery) to measure CD4 T cell profiles after *ex vivo* re-stimulation of splenocytes with SARS-CoV-2 trimer antigen (2 µg/ml cell culture medium incubated for 72 h at 37 °C and 5% CO_2_). The plates were analysed on a Sector Imager 2400 system (Meso Scale Discovery) and calculation of cytokine concentrations was performed by 4-parameter logistic non-linear regression analysis of the standard curve.

### Neutralization assay

Neutralization of SARS-CoV-2 pseudo-particles was evaluated using a pseudotyped lentivirus neutralization assay with SARS-CoV-2 spike (Wu-Hu-1) and HEK293T cells engineered to express human ACE2, as previously described.^21^ ID50 values were estimated by fitting a logistic curve in Prism 5 (GraphPad Software), bounded between 0% and 100%, and interpolating the dilution at which luciferase expression was reduced by 50% relative to wells in the absence of serum.

Neutralization of SARS-CoV-2 was performed using the SARS-CoV-2/human/Denmark/DK-AHH1/2020 isolate cultured in Vero E6 cells, as previously described.^24^^,^^25^ This isolate is closely related to the Wu-Hu-1 sequence (with E309K and D614G substitutions in the spike protein). For cross-neutralization studies, a B.1.617.2 delta-variant (DK-AHH3 isolate) and a B.1.1.529 omicron-variant (DK-AHH4 isolate) were used. In brief, SARS-CoV-2 at a multiplicity of infection (MOI) of 0.01 to 0.15 was incubated 1h at room temperature with serially diluted heat inactivated (at 56 °C for 30 min) plasma (1/100 to 1/51200 dilution) at a 1:1 ratio. Following incubation, plasma/virus mixtures were added to naïve Vero E6 cells in quadruplicates in 96-well plates. After incubation for 48 h at 37 °C and 5% CO_2_, cells were fixed and stained as described,^24^ but using SARS-CoV-2 spike chimeric monoclonal primary antibody (( 40150-D004, Sino Biological) and F(ab')2-goat anti-human IgG Fc cross-adsorbed secondary antibody conjugated to HRP (A24476, Invitrogen) for the B.1.617.2 variant and anti-SARS-CoV-2 spike rabbit polyclonal primary antibody (no. ab272504, Abcam) and goat anti-Rabbit IgG (H+L) secondary antibody conjugated to HRP (no. 31460, ThermoFisher) for the B.1.1.529 variant). Single spots representing virus infected cells were counted by an Immunospot series 5 UV analyser, and percent neutralization calculated. Neutralization curves were constructed and the ID50 of plasma was calculated using non-linear regression (Log [inhibitor] vs normalized response [variable slope]), using GraphPad Prism. A mouse derived SARS-CoV-2 spike neutralizing antibody (Sino Biological #40592-MM57, RRID: AB_2857935) was the positive control. Four replicates of a 1/800 dilution (1.25 µg/mL) of a mouse derived SARS-CoV-2 spike neutralizing antibody (Sino Biological #40592-MM57, RRID: AB_2857935) was used as positive control for each plate, giving an average of 81% neutralization for the homologous Wu-Hu-1 variant and 96% for the B.1.617.2 variant. A 1/80 dilution of plasma from a COVID-19 vaccinated individual from the Clinical, Virological and Immunological (CVIC) study^25^^,^^26^ was used in the same conditions for the B.1.1.529 variant, giving an average of 95% neutralization. The investigators performing the neutralization assays were blinded to the experimental groups.

### Meso Scale discovery ACE2 competition assay

The V-PLEX SARS-CoV-2 Panel 19 (ACE2) Kit was run according to the manufacturer's instructions. Briefly, serum samples (diluted x25, x250 and x2500) and nasal washes (diluted x5) were added to pre-coated V-plex plates followed by ACE2 binding detection using a Sector Imager 2400 system (Meso Scale Discovery).

### SARS-CoV-2 challenge in Syrian hamsters

Index hamsters were anesthetized with isofluorane and inoculated i.n. with 1.8 × 10^5^ TCID_50_ in 50 µl of the Danish SARS-CoV-2 isolate SARS-CoV-2/Hu/DK/SSI-H5 (GenBank accession number: ON809567). This isolate is closely related to the Wu-Hu-1 sequence (with V367F and E990A substitutions in the spike protein and a G251V substitution in ORF3a). In the transmission model each infected hamster was isolated for six hours and then placed in a cage containing one hamster immunized s.c/s.c., one immunized s.c./i.n. and one unvaccinated control (six cages in total). In the onwards transmission model, index hamsters were isolated for 24 h and then housed with s.c./i.n. vaccinated or unvaccinated contacts. 24 h later the contacts were co-housed with another set of naïve hamsters for the rest of the study (five days) to assess onwards transmission. Weight and signs of disease (lethargy, ruffled fur, hunched posture) were monitored until the study was terminated at seven days post infection of the index hamsters. Humane endpoints included weight loss >20% (none of the animals reached humane endpoints). Virus titres were determined in nasal washes at days two and seven post challenge and in lungs at the time of termination.

### RT-qPCR for SARS-CoV-2 detection

Lungs were dissociated by GentleMACS (M-tubes, Miltenyi). Nasal washes were collected by flushing with 350 µl of PBS. Total RNA extractions were done by MagNa Pure 96 system (Roche Molecular Biochemicals, Indianapolis, Indiana, United States (US)), using MagNA Pure LC DNA Isolation kit I lysis buffer. All oligonucleotides were synthesized by Eurofins Genomics. RT-qPCR was performed using 5 µl of resuspended RNA in a 25 µl reaction volume using the Luna® Universal Probe One-Step RT-qPCR with Luna WarmStart® RT Enzyme Mix (New England Biolabs) with 400 nM concentrations primers and 200 nM of probe. Primers and probes for the SARS-CoV-2 E gene target (diagnostic PCR) were as previously described.^27^ Cycling conditions were 55°C for 10 min, denaturation at 95 °C for 3 min, and 45 cycles of 95 °C (15 s) and 58 °C (30 s). To generate standard curves, synthetic SARS-CoV-2 RNA ctr1 (MT007544.1) (Twist Bioscience) of a known copy number was serially diluted. A stabilized RNA for 2019-nCoV E gene (EVAg) was used as positive control. RT-qPCR for detection of subgenomic RNA for the SARS-CoV-2 E-gene was performed as previously described^28^ using 50 °C for 10 min, 95 °C for 3 min, and 45 cycles of 95 °C (10 s), 56 °C (15 s) and 72 °C (5 s). Reactions were carried out using a Lightcycler-480 Real-Time PCR System (Roche). Results were expressed as log10-transformed numbers of genome equivalent copies per ml (nasal washes) or gram (lungs).

### Pathology

From animals euthanized on day seven post challenge, the right diaphragmatic lung lobe was pseudo-perfused fixed in 10% neutral buffered formalin and then placed in histo-cassettes for immersion fixation in 10% neutral buffered formalin for 24 h before being transferred to ethanol. Paraffin embedded tissues were sectioned (4 μm) and stained with haematoxylin and eosin (H&E) for histopathological examination. Sections were evaluated for infiltration of inflammatory cells (neutrophils and macrophages). In all sections the inflammatory reaction was scored as 0: absent, 1: few sporadic inflammatory cells present, and 2: numerous accumulated inflammatory cells. The inflammatory cells were identified from their characteristic morphology. The presence of type-II pneumocyte hyperplasia was determined from the presence of this cell type laying the alveolar lumen in a row morphology. Type-II pneumocyte hyperplasia was examined by H&E staining and confirmed by cytokeratin staining using a mouse anti-cytokeratin antibody (clone AE1/AE3, DAKO M3515). The investigators performing the histological examination were blinded to the experimental groups.

### Statistics

Differences between groups were analysed by one-way ANOVA comparing the mean of each column with each other column. Differences between naïve and s.c./i.n. immunized groups in the onward transmission model were analysed by unpaired student's t test (GraphPad v8.2.1). Sample size determination is described under immunizations.

### Role of funders

The funding source had no role in study design, collection, analysis or interpretation of data or in the writing of the publication.

## Results

### Parenteral prime – intranasal boost with adjuvanted SARS-CoV-2 spike trimer induces serum neutralizing antibodies and nasal IgA responses in mice

**Figure 1:**
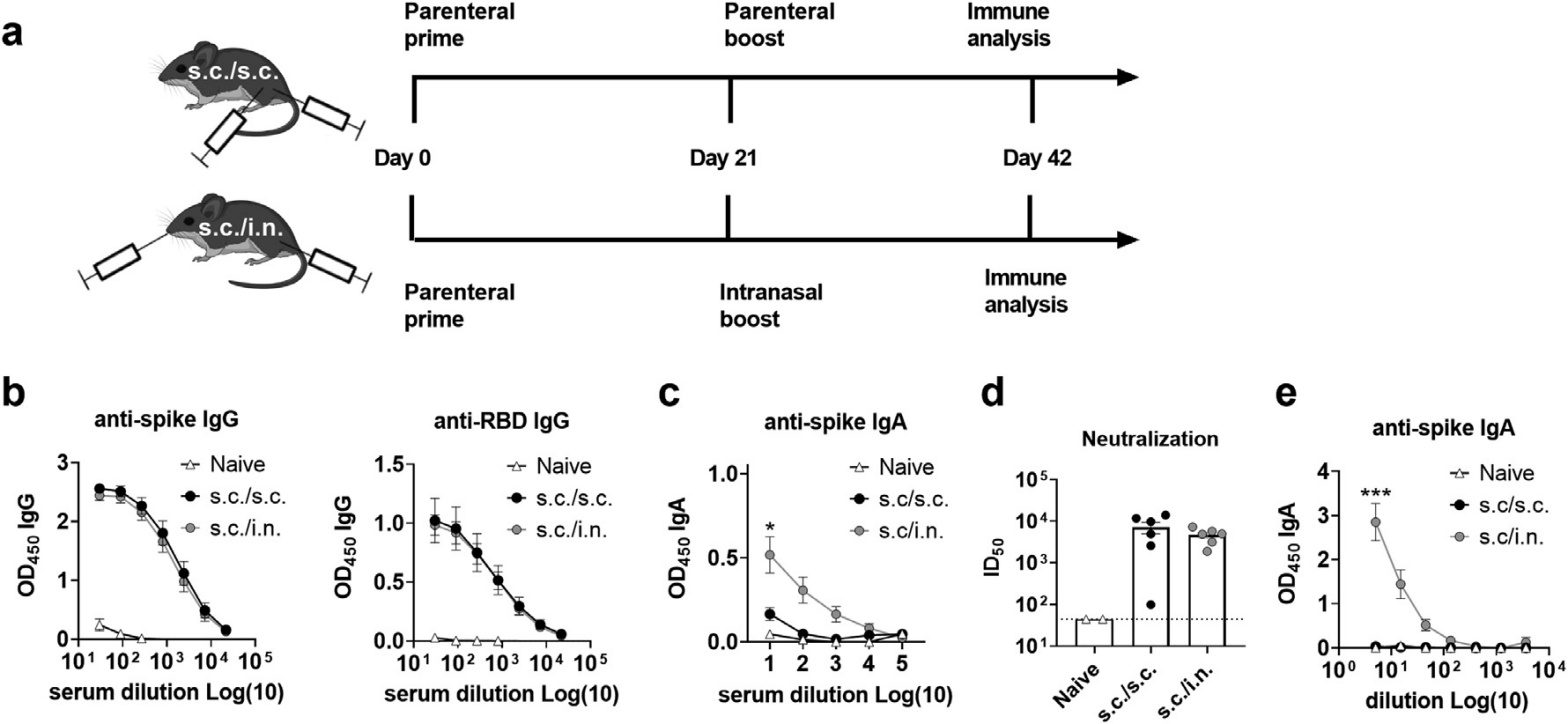
Mucosal boosting elicits serum neutralizing antibody responses comparable to parenteral immunization and additionally boosts IgA responses. Mice were immunized with two doses of spike trimer protein formulated in cationic liposomes (CAF®01). The trimer protein was either the S-2P version **(a-e)** or the HexaPro variant. **(f)** The vaccine was administered as a conventional subcutaneous two dose regimen (s.c./s.c.) or as subcutaneous priming followed by intranasal boosting (s.c./i.n.) Serum was sampled at 21 days after the 2^nd^ immunization. **a)** Experimental setup. **b)** IgG antibody responses against spike protein (left panel) and the receptor binding domain (RBD) (right panel). **c)** Serum IgA antibody responses against spike protein. **d)** Neutralization of SARS-CoV-2 by a pseudovirus neutralization assay. The stippled line indicates neutralization below the limit of detection and is plotted as ID50=45. **e)** Spike-specific IgA antibody responses in nasal washes. Mean ± SEM is displayed. Statistically significant differences are indicated by * or *** (one-way ANOVA, comparing the mean of each column with each other column, *p*<0.05 or 0.001, respectively). There was no statistically significance among groups if not otherwise indicated. Figures represent n= two (naïve controls) to six (vaccinated) mice per group. Created with BioRender.com.

Systemic neutralizing antibodies effectively protect the lower airways against SARS-CoV-2 pathology.^29^ We investigated if parenteral priming - i.n. boosting would induce similar or lower neutralizing antibody responses compared to administering the vaccine as a standard two-dose parenteral regimen. Prefusion-stabilized spike trimers containing two stabilizing proline mutations (S-2P)^18^ were formulated in a cationic liposome adjuvant (CAF®01) and characterised as described previously.^23^ The vaccine was tested in mice, administered as two s.c. immunizations (s.c./s.c.) or as one s.c. immunization followed by i.n. boost (s.c./i.n.) (Figure 1a). Serum anti-spike and anti-RBD IgG antibody levels at 21 days after the second immunization were similar between the two groups (Figure 1b), whilst spike-specific IgA responses were only detected in the s.c./i.n. group (Figure 1c). To investigate if the vaccine-elicited antibodies were capable of neutralizing SARS-CoV-2, we performed a homotypic SARS-CoV-2 pseudovirus neutralization assay and found similar neutralizing capacity of the sera (ID50 of 1/7222 for s.c./s.c. versus 1/4672 for s.c./i.n immunization) (Figure 1d). We also measured T cell responses in the spleen and found that s.c./i.n. immunization elicited similar systemic Th1 and Th17 responses as s.c./s.c. immunization (sFigure 1).

To investigate if i.n. boosting could facilitate a mucosal antibody response in the upper respiratory tract, we compared spike-specific IgA responses in nasal washes of s.c./i.n and s.c./s.c. immunized mice. For these studies we used the further stabilized Spike HexaPro trimer variant.^19^ Following formulation in CAF®01, the liposomes maintained a net positive charge and an intact binding of HexaPro spike to ACE2 was confirmed (sFigure 2). s.c./i.n immunization elicited significantly higher spike-specific IgA responses both in serum and in nasal washes compared to the s.c./s.c. regimen (*p*<0.001, one-way ANOVA) (Figure 1e). Thus, administering the booster immunization i.n. did not compromise systemic vaccine-elicited immunity, but facilitated IgA responses both systemically and in the upper airways.

**Figure 2:**
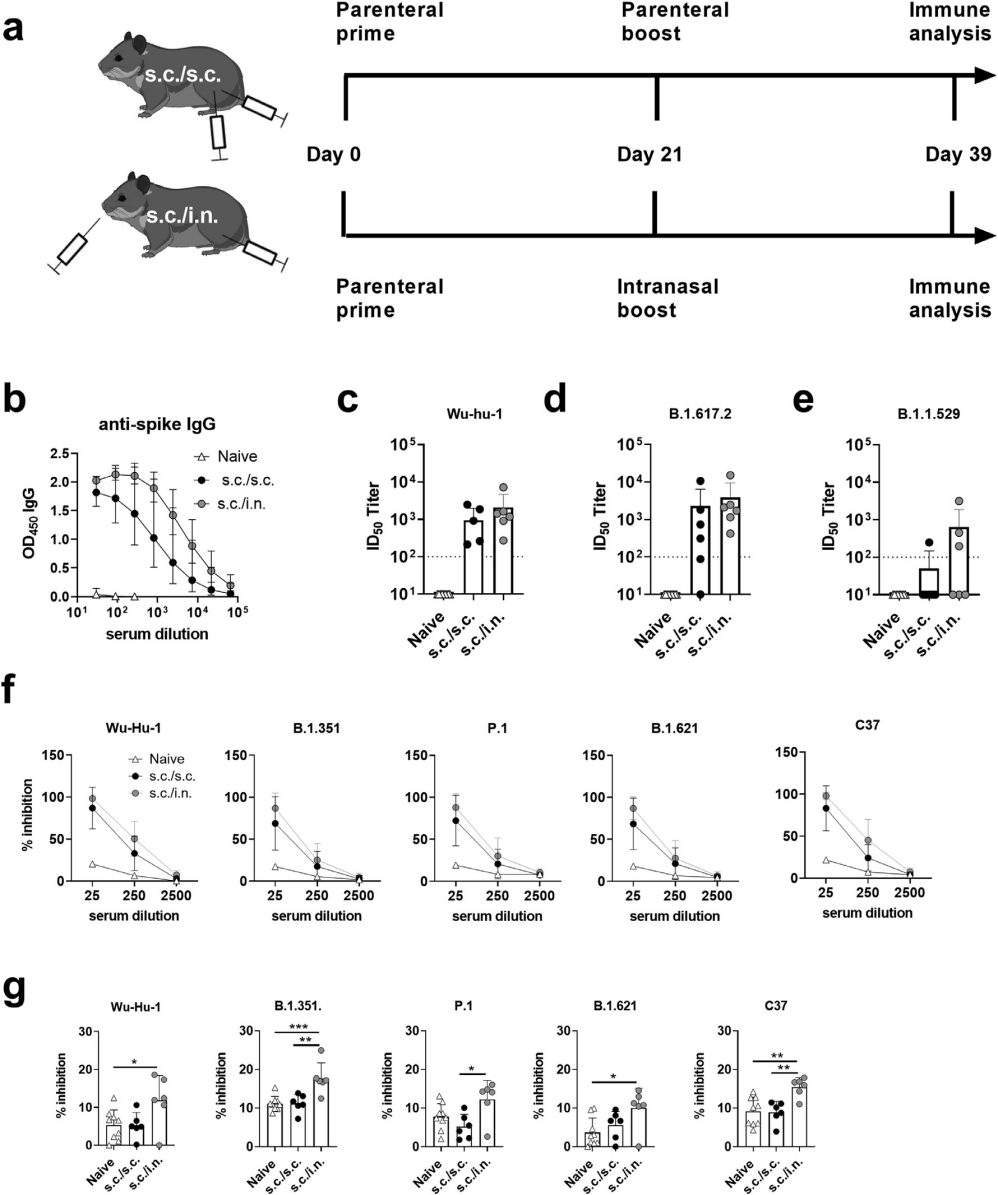
Syrian Hamsters were immunized with two doses of spike HexaPro trimer protein formulated in cationic liposomes (CAF®01). The vaccine was either administered as a subcutaneous two dose regimen (s.c./s.c.) or as subcutaneous priming followed by intranasal boosting (s.c./i.n). **a)** Experimental setup. **b)** Serum IgG antibody responses against spike protein (mean+95% CI). Serum neutralization of SARS-CoV-2 was tested in a culture derived SARS-CoV-2 assay against **c)** the homologous Wu-hu-1 strain and **d)** the delta variant (B.1.617.2). A SARS-CoV-2 spike neutralizing monoclonal antibody (40592-MM57) was used as positive control at 1/800 dilution, which gave an average of 81% neutralization for the homologous Wu-Hu-1 variant and 96% for the B.1.617.2 variant. **e)** Neutralization of the omicron variant (B.1.1.529). Plasma from a COVID-19 vaccinated individual (1/80 dilution) was used as positive control giving 95% neutralization. The stippled line indicates neutralization below the limit of detection and is plotted as ID50=100. **f)** ACE2 competition assay measuring serum antibodies towards the Wu-Hu-1, B.1.351, P.1, B.1.621 and C37 strains. **g)** ACE2 competition assay measuring nasal washes antibodies towards the Wu-Hu-1, B.1.351, P.1, B.1.621 and C37 strains. Bars indicate mean+SD. Statistically significant differences are indicated by *, ** or *** (one-way ANOVA, comparing the mean of each column with each other column, *p*<0.05, 0.01 or 0.001, respectively). There was no statistically significance among groups if not otherwise indicated. Figures represent n= nine unvaccinated (naïve) and six vaccinated hamsters per group. Created with BioRender.com.

### Intranasal booster vaccine protects against disease and reduces viral replication in the upper airways in a SARS-CoV-2 hamster transmission model

SARS-CoVs mainly transmit via aerosol or large droplet transmission. To investigate if i.n. booster vaccination could protect against virus transmission, we developed a model in Syrian hamsters reflecting natural SARS-CoV-2 transmission, where index hamsters were infected i.n. and co-housed with animals vaccinated either s.c./s.c or s.c./i.n. or left unvaccinated. Hamsters were immunized with an identical regimen as in the mouse studies, using pre-fusion stabilized Spike HexaPro trimer (Figure 2a). Similarly to what we observed in mice, s.c./i.n. vaccination in hamsters elicited comparable serum IgG responses (Figure 2b) and neutralizing antibody responses against the homologous strain (Wu-hu-1) as s.c./s.c. immunization, when measured in a replication competent virus neutralization assay (Figure 2c). Similar cross-neutralizing responses were also found against the delta (B.1.617.2) variant (Figure 2d), whilst only one of six animals in the s.c./s.c. immunized group and three of six in the s.c./i.n. immunized group had antibodies cross-neutralizing the omicron (B.1.1.529) variant (Figure 2e). To further probe neutralizing antibody responses against other SARS-CoV-2 variants, we used an ACE-2 binding competition assay. Both s.c./s.c and s.c./i.n. immunized animals had antibodies competing for ACE-2 binding of the beta (B.1.351), gamma (P.1), lambda (C37) and Mu (B.1.621) variants (Figure 2f). To test the capability of the two vaccine strategies to elicit antibody responses in the upper respiratory tract, nasal washes were isolated 21 days after s.c. or i.n. booster immunization and tested in the ACE-2 competition assay. Although responses were low in all groups, s.c./i.n. immunized hamsters had significantly higher responses than naïve animals against the homologous Wu-Hu-1 and the B.1.351, B.1.621 and C37 strains (*p*<0.05, one-way ANOVA) (Figure 2g). s.c./i.n. immunized hamsters also had higher responses than their s.c./s.c. immunized counterparts against the B.1.351, P.1 and C37 strains (*p*<0.05, one way ANOVA).

**Figure 3:**
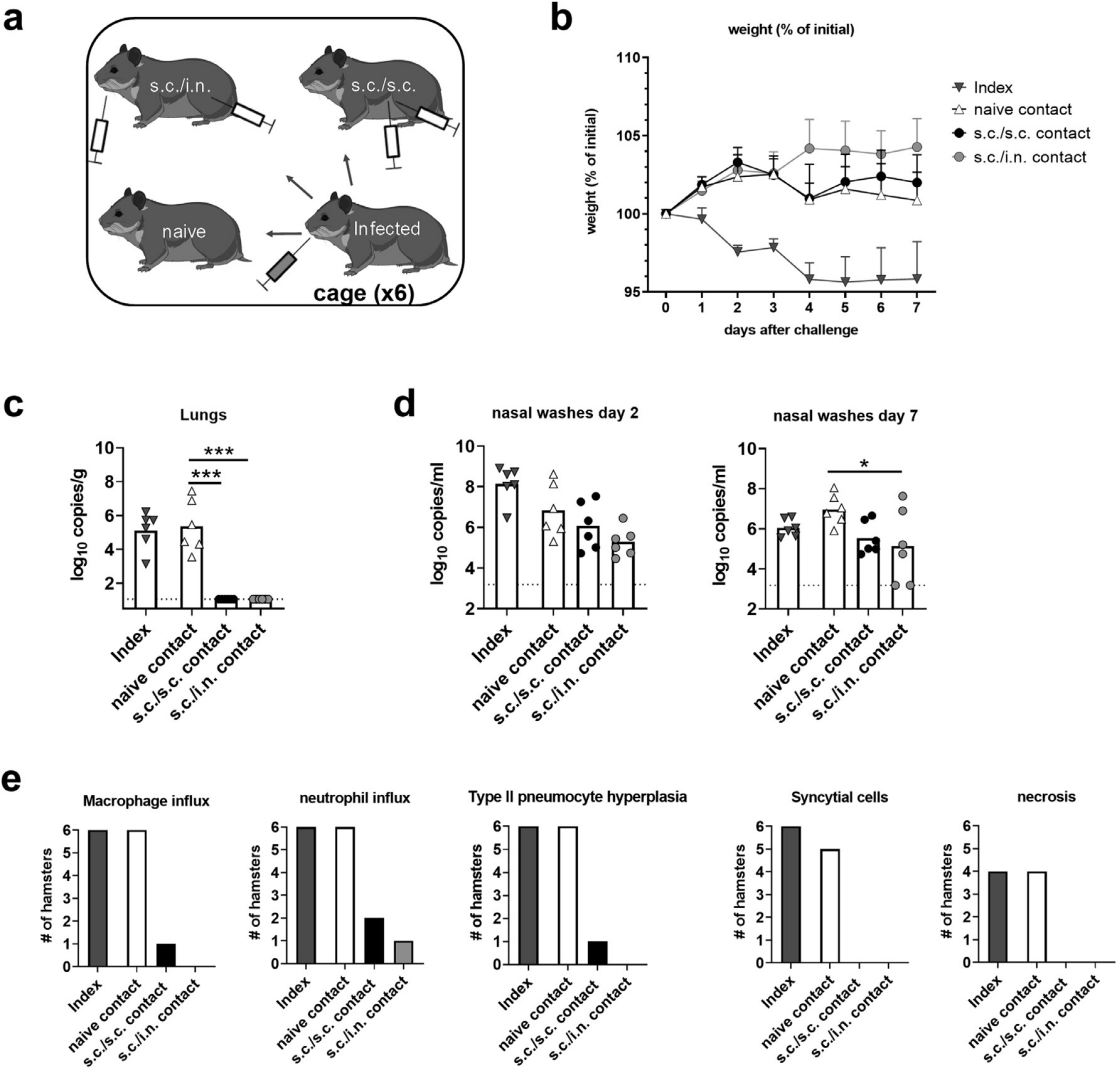
Syrian hamsters were vaccinated subcutaneously (s.c./s.c.) or via subcutaneous priming – intranasal boost (s.c./i.n) with spike HexaPro trimer protein formulated in cationic liposomes (CAF®01). The hamsters were housed with index animals, which had been challenged intranasally with 1.8 × 10^5^ TCID_50_ of SARS-CoV-2. **a)** Schematic of the study setup. **b)** Percent weight change (mean+SEM). **c)** Viral load in lungs at 7 dpi and **d)** nasal washes at 2 dpi and 7 measured by a diagnostic qPCR against the E-gene (bars indicate mean). e**)** Following fixation the right diaphragmatic lung lobe were fixed in 10% formalin, cut and stained with H&E to examine for pulmonary pathology at 7 dpi. Plots display the numbers of hamsters in each group having influx of the indicated inflammatory cells, type II pneumocyte hyperplasia, formation of syncytial cells and necrosis. (*n* = 6 hamsters per group). Statistically significant differences between contact animals are indicated by * or *** (one-way ANOVA, one-way ANOVA, comparing the mean of each column with each other column *p*<0.05 or 0.001, respectively). There was no statistically significance among groups if not otherwise indicated. Figures represent n= six hamsters per group. Created with BioRender.com.

The hamsters were challenged with SARS-CoV-2 at 21 days after the 2^nd^ immunization. Animals were housed in six cages each containing one s.c./s.c and one s.c./i.n. immunized animal together with two unvaccinated animals. One of the latter was placed in a separate cage and challenged i.n. with 1.8 × 10^5^ TCID_50_ of the SARS-CoV-2/Hu/DK/SSI-H5 isolate (index hamster) (Figure 3a). Six hours later, the index hamsters were returned to their original cages. Index hamsters and contacts were then followed for seven days to measure virus transmission and signs of disease. Only the infected index animals had marked weight loss (Figure 3b). However, at day seven post challenge, viral RNA was detected in the lungs of both index animals and unvaccinated contacts by a diagnostic PCR for the E-gene (Figure 3c). In contrast, no viral RNA was detected in the lungs of vaccinated animals (both after s.c./s.c. and s.c./i.n. immunization). In the upper airways, viral RNA was detected in all groups, although there was a tendency towards lower levels in nasal washes from contacts vaccinated s.c./i.n. compared to unvaccinated controls at two days post challenge (not significant, *p* =0.06, one-way ANOVA) and significantly lower levels at day 7 post challenge (*p*<0.05) (Figure 3d). There was no statistically significant differences in viral load between s.c./s.c. and s.c./i.n. immunized animals. Infection with the Wu-hu-1 strain further boosted neutralizing antibody titres in both the s.c./s.c. and s.c./i.n. immunized groups against both the homologous strain and the delta and omicron variants (sFigure 3). Infection per se only elicited detectable neutralizing antibodies against the Wu-hu-1 strain and the delta variant, but not the omicron variant (sFigure 3). Overall, these results demonstrated that the s.c./s.c. and s.c./i.n. vaccine elicited similar systemic neutralizing antibody responses and both immunization strategies effectively protected against SARS-CoV-2 in the lower airways. In addition, s.c./i.n. immunization elicited antibody responses in the nasal cavity and partially protected against virus in the upper airways.

**Figure 4:**
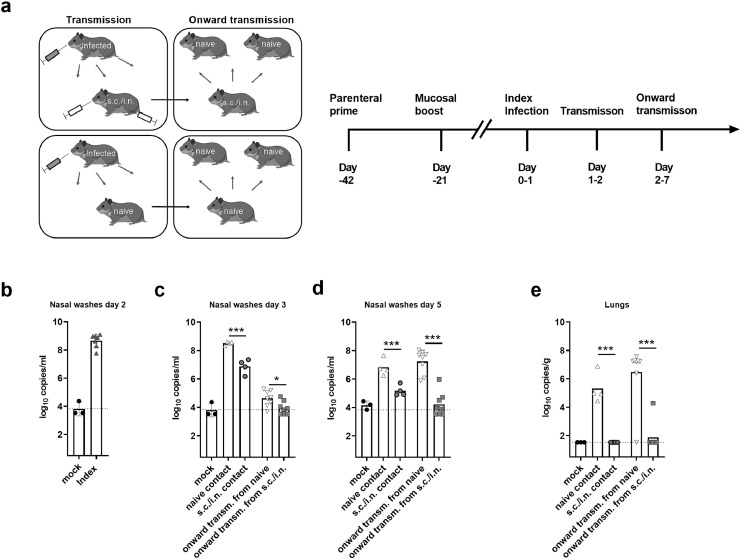
Syrian hamsters were vaccinated with spike HexaPro trimer protein formulated in cationic liposomes (CAF®01) via subcutaneous priming – intranasal boost (s.c./i.n) or left unvaccinated (naïve). The hamsters were then housed with index animals, which had been challenged intranasally with 1.8 × 10^5^ TCID_50_ of SARS-CoV-2 24 h earlier. Virus transmission was allowed to occur for 24 h. The vaccinated and unvaccinated hamsters were then co-housed for five days with another set of naïve animals to monitor onward transmission. **a)** Schematic of the study setup. **b)** Viral load in nasal washes of index animals at two days post infection (dpi) measured by a diagnostic qPCR against the E-gene **c)** Viral load in nasal washes of contact animals measured at 3 dpi and **d)** 5 dpi measured by a diagnostic qPCR against the E-gene. **e)** Viral load in lungs at 7 dpi. The stippled lines indicate the limit of detection. Bars indicate mean. Figures represent *n*= three (mock), four (index and vaccinated) or eight (onward contacts) hamsters per group. There was no statistically significance among groups if not otherwise indicated. Statistically significant differences are indicated by *** (Student t-test, *p*<0.01). Created with BioRender.com.

SARS-CoV-2 causes lung pathology in hamsters, including infiltration with neutrophils and mononuclear cells and hyperplasia of type-II pneumocytes.^30^^,^^31^ To examine if the vaccines could protect against pulmonary pathology, hamsters were examined for lung pathology at seven days post infection. Index animals and unvaccinated contacts had influx of neutrophils and macrophages into the alveolar tissue (Figure 3e and sFig4a-b). This was accompanied by marked type II pneumocyte hyperplasia and syncytial cell formation (Figure 3e and sFigures4c-d). Four of six animals in the unvaccinated contact group also had necrosis of alveolar tissues. Of vaccinated animals, one in the s.c/s.c. that had moderate pulmonary inflammation with macrophage and neutrophil influx and presence of syncytial cells, whilst another animal had mild inflammation with neutrophil influx. In the s.c/i.n. group, only one hamster had mild pulmonary inflammation with neutrophil influx but no syncytial cells observed (Figure 3e and Figure 4). Thus, both s.c./i.n. and s.c./s.c. immunization with CAF®01-adjuvanted spike HexaPro vaccine effectively protected against lung pathology in the hamster model.

### Parenteral prime - intranasal booster vaccine protects against onward transmission

Apart from protecting the individual, a major goal of vaccination is to prevent virus transmission, thereby protecting against virus spread in the population. To study the impact of vaccination on transmissibility of SARS-CoV-2, we refined the hamster transmission model to assess onward virus transmission from vaccinated animals (Figure 4a). Previous studies in Syrian golden hamsters found that the highest frequency of transmission to contacts occurred at 16–48 h post infection, which was the time of peak viral load in the donor animals.^32^ Index hamsters were therefore infected with 1.8 × 10^5^ TCID50 of SARS-CoV-2 and isolated for 24 h and were then co-housed with either vaccinated animals, having received parenteral prime - i.n. booster vaccine, or naïve animals for another 24 h to allow virus transmission. The index hamsters had confirmed SARS-CoV-2 by a diagnostic qPCR at day 2 post infection (Figure 4b). To study if the parenteral prime – mucosal boost vaccine protected against onward transmission, the vaccinated or naïve contacts were subsequently co-housed with another set of naïve hamsters. qPCR of nasal washes sampled 24 h later (Day 3), revealed virus in all s.c/i.n. vaccinated hamsters, although virus titres were significantly lower than in naïve (unvaccinated) controls (*p*<0.001, unpaired t-test) (Figure 4c). In contrast, both of the onward transmission groups had virus loads at the detection limit at this time point. At 48 h later (day 5), extensive onward transmission had occurred from naïve contacts, whilst naïve animals co-housed with the vaccinated group had significantly lower virus levels, still approaching the limit of detection (*p*<0.001) (Figure 4d). Onward transmission was also evident in the lungs of all naïve hamsters co-housed with the unvaccinated controls, whilst only one of eight animals co-housed with the s.c./i.n. immunized hamsters had detectable viral RNA in the lungs (significantly lower than in the naïve contacts, *p*<0.001, unpaired t-test) (Figure 4e). Another qPCR for subgenomic RNA indicated that the virus detected in the upper airways of s.c./i.n. immunized was replication-competent at days 3 and 5 into the study, but levels were significantly lower than in the unvaccinated group (*p*<0.05, unpaired t-test) (sFigure 5). Thus, although s.c./i.n. immunization did not provide sterilizing immunity in the upper respiratory tract, the vaccine effectively protected against onward transmission.

## Discussion

SARS-CoV-2 has caused more than 6 million deaths to date. Safe and effective vaccines against SARS-CoV-2 have been produced and distributed at unprecedented speed and have completely changed the course of the pandemic. Vaccines have proven extremely effective to date at reducing morbidity and mortality. However, with continued onward transmission, there is still a risk that novel variants of concern (VOC) emerge, necessitating continued development of novel vaccines. Furthermore, improved vaccination strategies must be explored to prepare for future pandemics caused by respiratory viruses. One major drawback of licensed SARS-CoV-2 vaccines is the relatively low protection induced in the upper airways,^29^^,^^33^ which may allow for virus replication and onward transmission. To protect against emerging viruses with pandemic potential, a vaccine approach that can effectively prevent virus transmission is desired. One key strategy to this aim is via induction of mucosal immune responses in the upper airways. Whilst parenteral vaccines provide only low immunity at this site, vaccines applied in the nasal cavity can induce a number of immune mediators that can block viral entry or virus replication, including sIgA^6^ and local nasal-associated lymphoid tissue (NALT) CD4 and CD8 T cell responses.^34^ In the present study we tested a combination of parenteral priming with mucosal boosting to elicit both systemic and local airway immunity and evaluated this in a Syrian hamster transmission model.

Syrian hamsters are a highly relevant model for human SARS-CoV-2 infection, displaying similar viral kinetics, pathological signs and disease course as seen in patients with COVID-19.^31^^,^^35^ Previous studies have demonstrated that hamsters immunized i.n. with chimpanzee adenovirus encoding prefusion-stabilized spike protein had reduced virus titres in the upper respiratory tract compared to unvaccinated controls.^36^ Similarly, hamsters immunized i.n. with the adenovirus-vectored vaccine ChAdOx1 nCoV-19/AZD1222 had significantly lower virus titres in the upper airways than unvaccinated controls, after being co-housed with SARS-CoV-2 infected animals.^37^ A recombinant spike-based protein vaccine linked to outer membrane vesicles (OMV) was also found to protect hamsters against pathology after i.n. administration, but did not reduce virus titres in nasal turbinates.^38^ Here we have demonstrated that the prefusion stabilized (HexaPro) SARS-CoV-2 spike protein,^19^ adjuvanted with CAF®01 and given as parenteral prime – i.n. boost protects against lower respiratory tract infection and pathology upon SARS-CoV-2 challenge in Syrian hamsters. Furthermore, the vaccine significantly reduced viral load in the upper airways. It is possible that other vaccine strategies, such as parental prime followed by a mucosal boost with a heterologous vaccine,^39^ would be even more beneficial at inducing mucosal immunity. As application of the mucosal booster vaccine in humans would require special devices, e.g. nasal spray syringe or nebulizer, the methods of delivery could also influence the effectiveness of mucosal booster strategies.

A major goal of vaccination in a pandemic scenario is to protect against onwards transmission, thus limiting spread of virus in the population. We therefore developed a model in Syrian hamsters designed to investigate onwards transmission from vaccinated animals. Interestingly, we observed little onward transmission from hamsters vaccinated via parenteral prime – i.n. boost, although all the vaccinated animals had detectable virus in the upper airways. Even though we did not directly measure infectious titres in the current study, it is possible that a certain viral load in the upper airways is required for effective transmission, which would be surprising since previous studies have demonstrated that hamsters are highly susceptible to SARS-CoV-2, requiring only a few infectious particles to infect.^40^ Another intriguing possibility is that virus may be kept at bay by vaccine-induced mucosal antibodies, thereby preventing transmission. Since detection of virus in the upper respiratory tract has been directly associated with transmission, our studies indicating that virus in the upper respiratory tract of mucosally vaccinated animals does not readily transmit, therefore raises the question if detection of SARS-CoV-2 in the upper respiratory tract is necessarily indicative for risk of onwards transmission. Several SARS-CoV-2 vaccine strategies, including i.n. vaccine regimens, have demonstrated complete protection against SARS-CoV-2 in the lower airways, but incomplete clearance in the upper airways.^36^^,^^37^^,^^41^^,^^42^ To what extent these strategies protect against onwards transmission is an important question, which should be further studied for different pandemic vaccine candidates. An important limitation of the current study is that, although parenteral prime – i.n. boost protected against onward transmission, we did not compare this regimen head-to-head with parenterally administered vaccines. Future studies should therefore include parenteral only vaccines, e.g. the licensed NVX-CoV2373^43^ or Comirnaty^®3^, in the onward transmission model to gauge the benefit of parenteral prime-intranasal boost over standard parenteral vaccines.

Despite the promise of i.n. vaccines to offer localized mucosal immunity in the upper airways, only few i.n. vaccines are registered, including the Flumist® vaccine against influenza. Parenteral prime – i.n. boost has been demonstrated to elicit strong and protective mucosal immune responses in a number of disease settings^11^^,^^39^^,^^44^ and proof-of-concept has also been demonstrated in humans where volunteers vaccinated parenterally with cationic liposome (CAF®01) adjuvanted *Chlamydia trachomatis* subunit vaccine and boosted i.n. with antigen alone, developed mucosal IgA antibody responses in the airways and the female reproductive tract.^15^ Although unadjuvanted protein given i.n. can boost parenterally primed responses (e.g. after mRNA-LNP or protein in adjuvant immunization),^15^^,^^45^ a more feasible strategy, particularly in a future pandemic mass-vaccination setting, would be to use similar vaccine formulations for both the systemic priming and mucosal boosting. We demonstrate here that spike protein formulated in CAF®01 and given as a parenteral prime - i.n. boost regimen completely protected hamsters against lung pathology following SARS-CoV-2 infection. Furthermore, the vaccine reduced virus loads in the upper airways and protected against onward transmission, thus highlighting the promise of this approach for vaccination against respiratory viruses of pandemic potential.

## Contributors

D.C., C.P., G.M., B.M., H.E.J., G.J., K.I., L.K.I., I.R., F.F., J.B. and G.K.P. designed research D.J.S., L.H., A.M.M., K.T.H., R.F.J., J.S.H., C.F., S.R. performed experiments D.C., C.P, H.E.J., K.T.H., R.F.J., J. S. H., C.F., S.R. and G.K.P. analyzed data. D.C., J.B., G.K.P. wrote the paper. All authors read and approved the final version of the manuscript, and have had access to the raw data. D.C and G.K.P. can verify the accuracy of the raw data for the study.

## Data sharing statement

Study protocol and all data collected for the study, including raw data and data analysis will be made available to others upon request. All data will be available upon publication of the manuscript, by contacting the corresponding author. Data will be made available after approval of a proposal and with a signed data access agreement.

## Declaration of interests

D.C. is co-inventor on patents on the cationic adjuvant formulations (CAF). All rights have been turned over to Statens Serum Institut, which is a non-profit government research facility. The rest of the authors declare that there are no competing interests.

## Appendix Supplementary materials

Image, application 1

Image, image 2

Image, image 3

Image, image 4

Image, image 5

Image, image 6
