# Supplementary material for: Protection against SARS-CoV-2 transmission by a parenteral prime—Intranasal boost vaccine strategy
Source: eBioMedicine. 2022 Sep 7;84:104248. doi: 10.1016/j.ebiom.2022.104248 (PMC9448948; doi:10.1016/j.ebiom.2022.104248)
Supplement: Supplementary file 1 [file mmc1.docx]

**sFig 1**

Mice were immunized with two doses of spike trimer S-2P protein formulated in cationic liposomes (CAF^®^01). The vaccine was administered as a conventional subcutaneous two dose regimen (s.c./s.c.) or as subcutaneous priming followed by intranasal boosting (s.c./i.n.). Systemic T cell responses were measured by stimulating splenocytes with full spike S-2P protein and assaying IFN-γ, IL-17, IL-5, IL-13 and IL-10 in the supernatant. Figures represent n= two (naïve controls) to six (vaccinated) mice per group

**sFig 2**

Characterization of cationic liposomes (CAF^®^01) formulated with spike HexaPro. **a)** The particle size (left panels), polydispersity index (PDI, middle panels) and zeta potentials (Zp, right panels) of the adjuvant formulations were tested by laser-Doppler electrophoresis. **b)** Binding of the vaccine formulation (spike HexaPro in CAF^®^01) to ACE2 was tested by ELISA.

**sFig 3**

Syrian Hamsters were immunized with two doses of spike trimer protein (hexaPro) formulated in cationic liposomes (CAF^®^01). The vaccine was either administered as a subcutaneous two dose regimen (s.c./s.c.) or as subcutaneous priming followed by intranasal boosting (s.c./i.n). Three weeks after the second immunization, the index animals were challenged intranasally with 1.8x10^5^ TCID_50_ of SARS-CoV-2 and co-housed with the vaccinated animals or naïve controls for 7 days. Neutralization of SARS-CoV-2 was tested in a homotypic culture derived SARS-CoV-2 assay pre-infection and day 7 post challenge against **a)** the homologous Wu-hu-1 strain **b)** the delta variant (B.1.617.2) and **c)** the omicron variant (B.1.1.529). A SARS-CoV-2 spike neutralizing monoclonal antibody (40592-MM57) was used as positive control at 1/800 dilution, which gave an average of 81% neutralization for the homologous Wu-Hu-1 variant and 96% for the B.1.617.2 variant. Plasma from a COVID-19 vaccinated individual (1/80 dilution) was used as control for the B.1.1.529 variant, giving 95% neutralization. Figures represent n= six hamsters per group.

**sFig 4**

Index hamsters were challenged intranasally with 1.8x10^5^ TCID_50_ of SARS-CoV-2. After 24 hours, the index hamsters were placed in cages with animals vaccinated twice subcutaneously (s.c./s.c.), by parenteral priming – intranasal boost (s.c./i.n.) or unvaccinated controls (naïve) for six days. Hamsters were sacrificed at seven days post infection of the index hamsters (related to figure 3). **a)** Severity score for pulmonary inflammation from individual hamsters (n=6). Each animal was scored as 0: absent, 1: few sporadic inflammatory cells present, and 2: numerous accumulated inflammatory cells (macrophages and neutrophils). **b)** Representative images of H&E stained of lungs from contact animals of SARS-CoV-2-infected hamsters. Lesions with significant influx of neutrophils and macrophages together with type II pneumocyte hyperplasia and syncytial cell formation were predominantly observed in alveolar tissue of naïve contact animals. The scale bars represent 90 µm (upper row) and 35 µm (lower row). **c)** Left panel: Representative staining showing identification of of type II pneumocytes present together with inflammation due to infiltration of neutrophils and macrophages (encircled area). C = Syncytial cells. H & E stain. X 10. Right panel: The infiltrating inflammatory cells, i.e. macrophages (M) and neutrophils (N), are present together with syncytial cells (S) and hyperplasia of type II pneumocytes (encircled). H & E stain. X 25 **d)** Immunostaining for cytokeratin confirms the hyperplasia of type II pneumocytes (Tp) and the epithelial origin of syncytial cells (C). X 25.

**sFig 5**

Syrian Hamsters were immunized with two doses of spike trimer protein (HexaPro) formulated in cationic liposomes (CAF^®^01). The vaccine was either administered as a subcutaneous two dose regimen (s.c./s.c.) or as subcutaneous priming followed by intranasal boosting (s.c./i.n). Three weeks after the second immunization, the index animals were challenged intranasally with 1.8x10^5^ TCID_50_ of SARS-CoV-2 and co-housed with the vaccinated animals or naïve (unvaccinated) controls for 1 day. The vaccinated and unvaccinated hamsters were then co-housed for five days with another set of naïve animals to monitor onward transmission**.** Nasal washes were isolated and tested by qPCR for subgenomic RNA of the E gene at **a)** day 2**, b)** day 3 and **c)** day 5 post infection of the index hamsters. Statistically significant differences are indicated by *, ** or *** (Student t-test, p<0.05, 0.01 or 0.001, respectively). There was no statistically significance among groups if not otherwise indicated. Figures represent n= three (mock), four (index and vaccinated) or eight (onward contacts) hamsters per group.
