# Supplementary figures and images for: Protection against SARS-CoV-2 transmission by a parenteral prime—Intranasal boost vaccine strategy
Source: eBioMedicine. 2022 Sep 7;84:104248. doi: 10.1016/j.ebiom.2022.104248 (PMC9448948; doi:10.1016/j.ebiom.2022.104248)

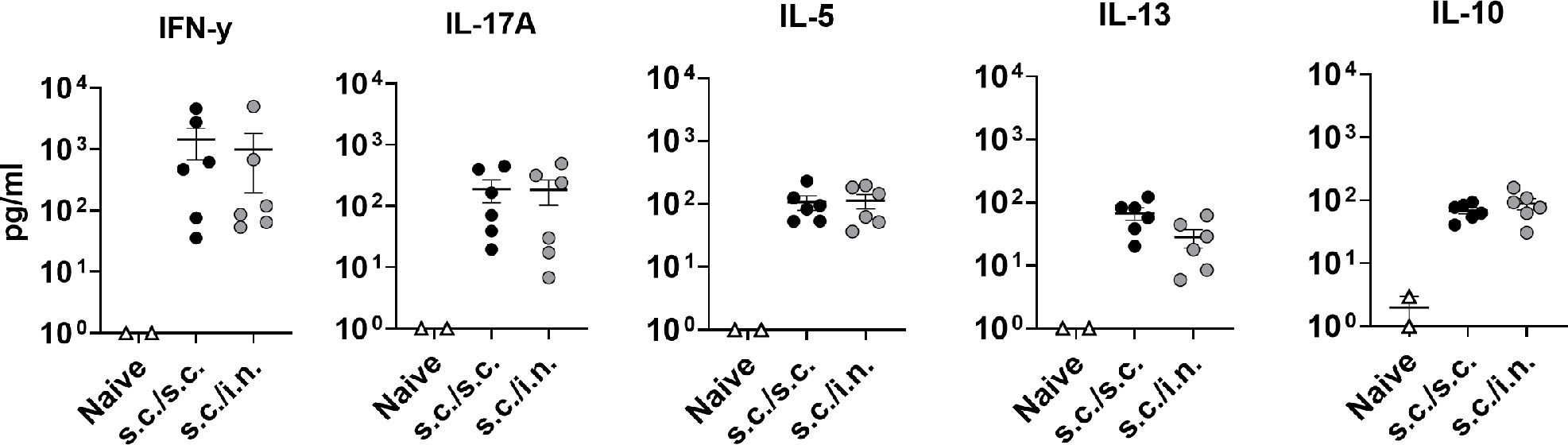

Supplement: Supplementary file 2 [file mmc2.jpg]

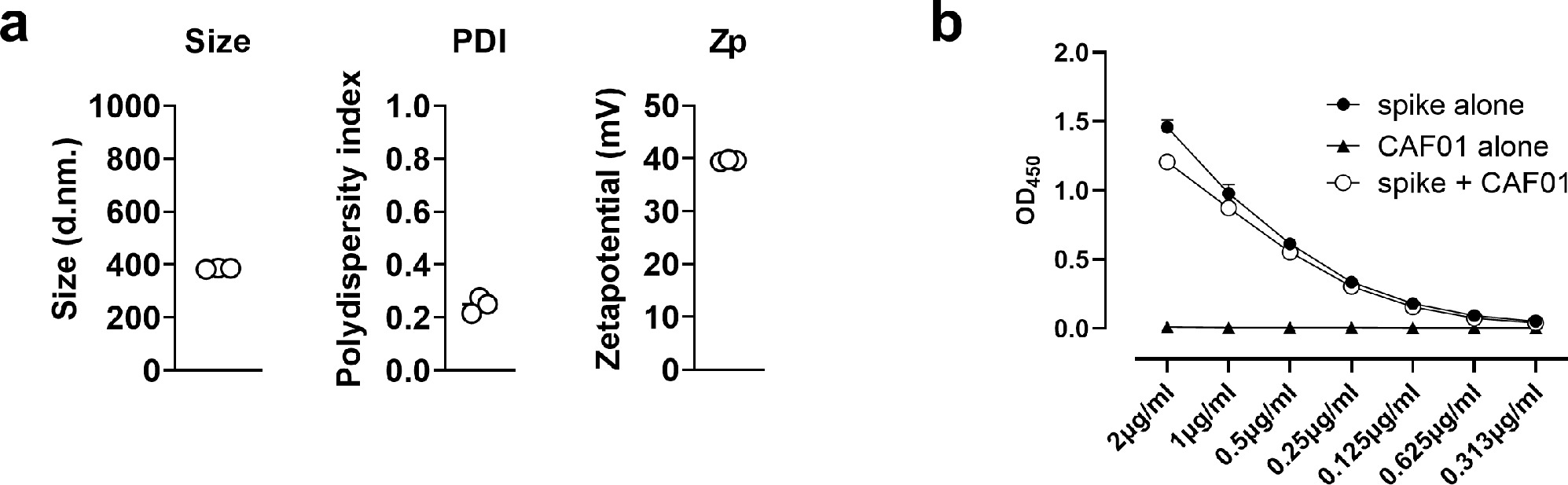

Supplement: Supplementary file 3 [file mmc3.jpg]

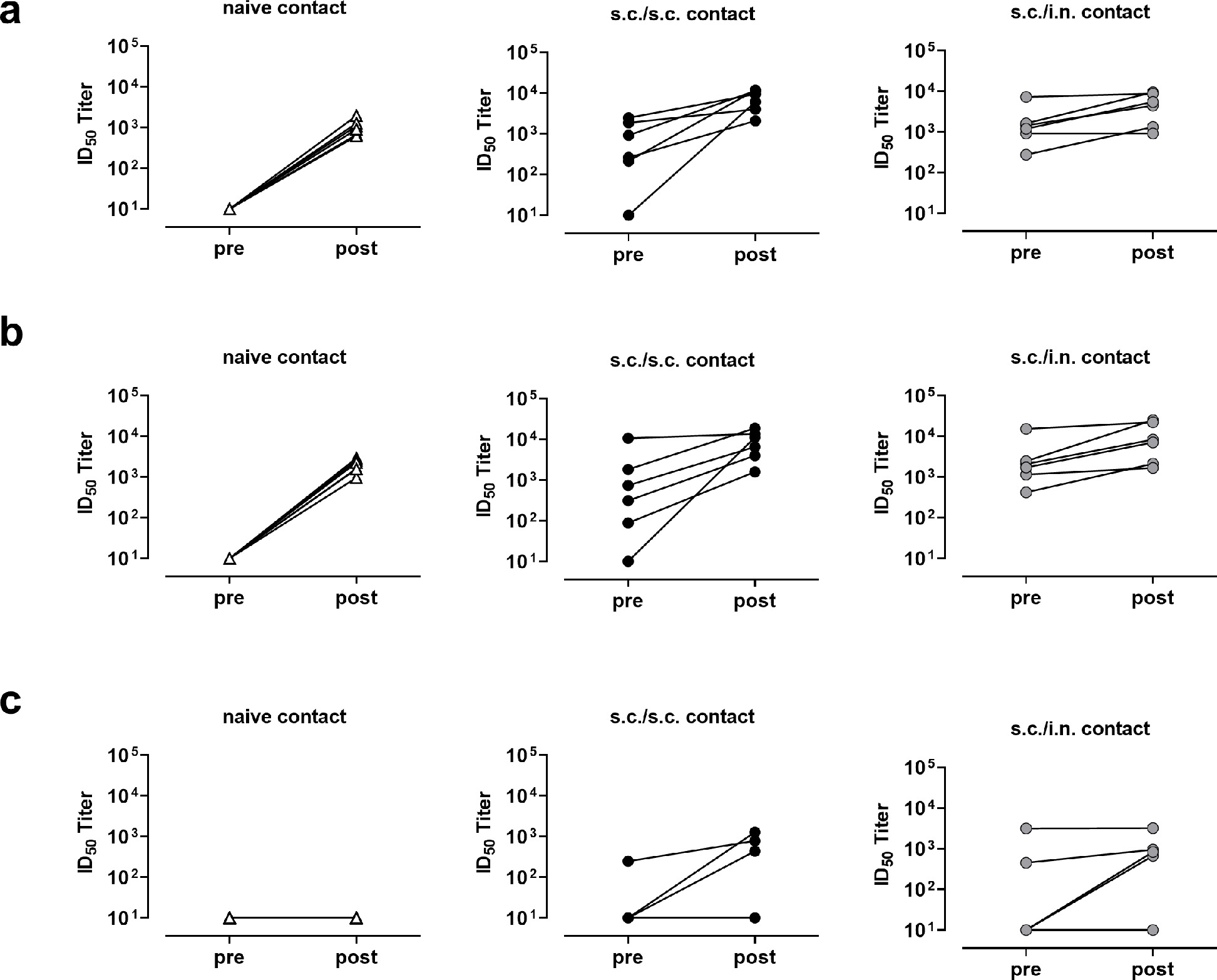

Supplement: Supplementary file 4 [file mmc4.jpg]

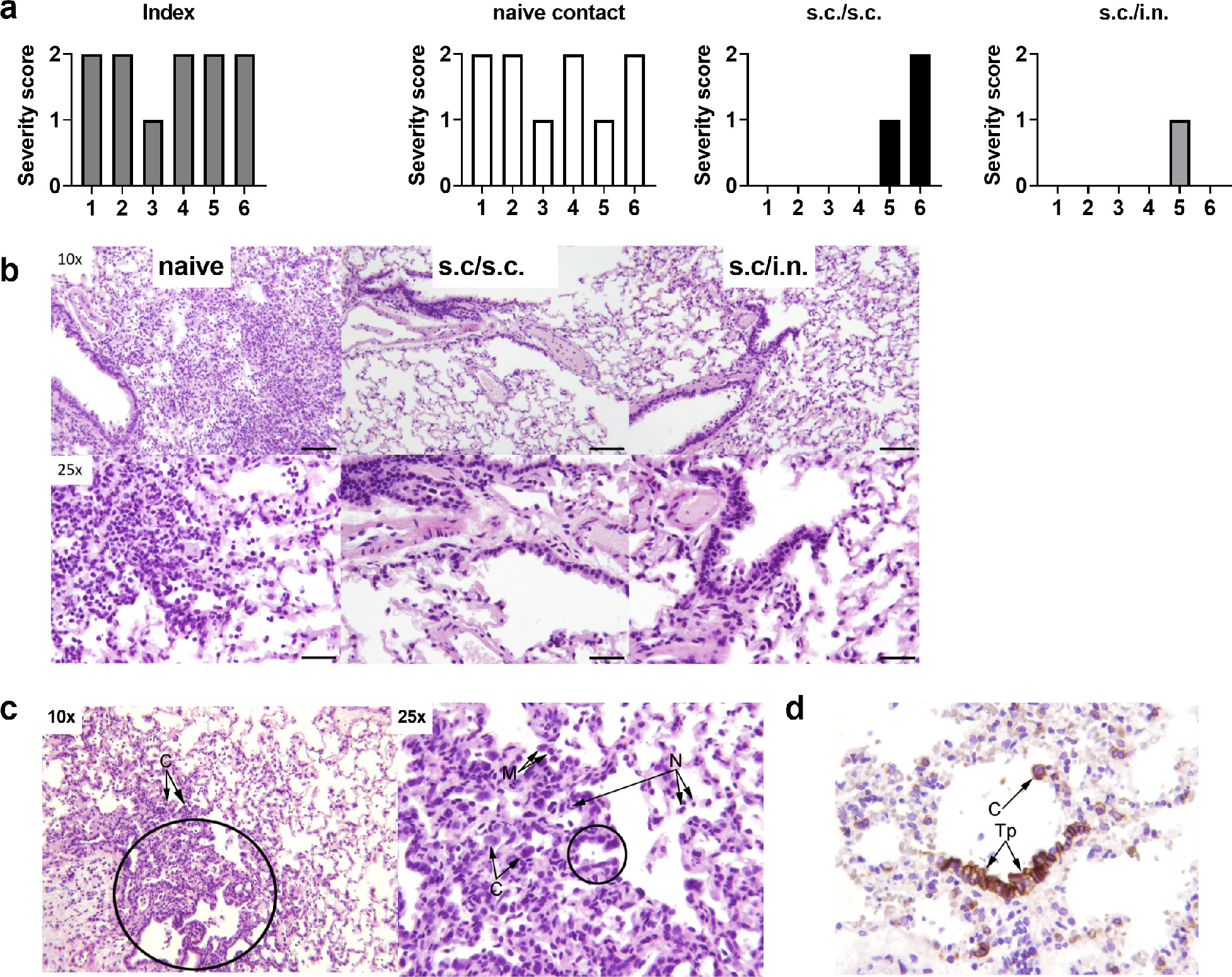

Supplement: Supplementary file 5 [file mmc5.jpg]

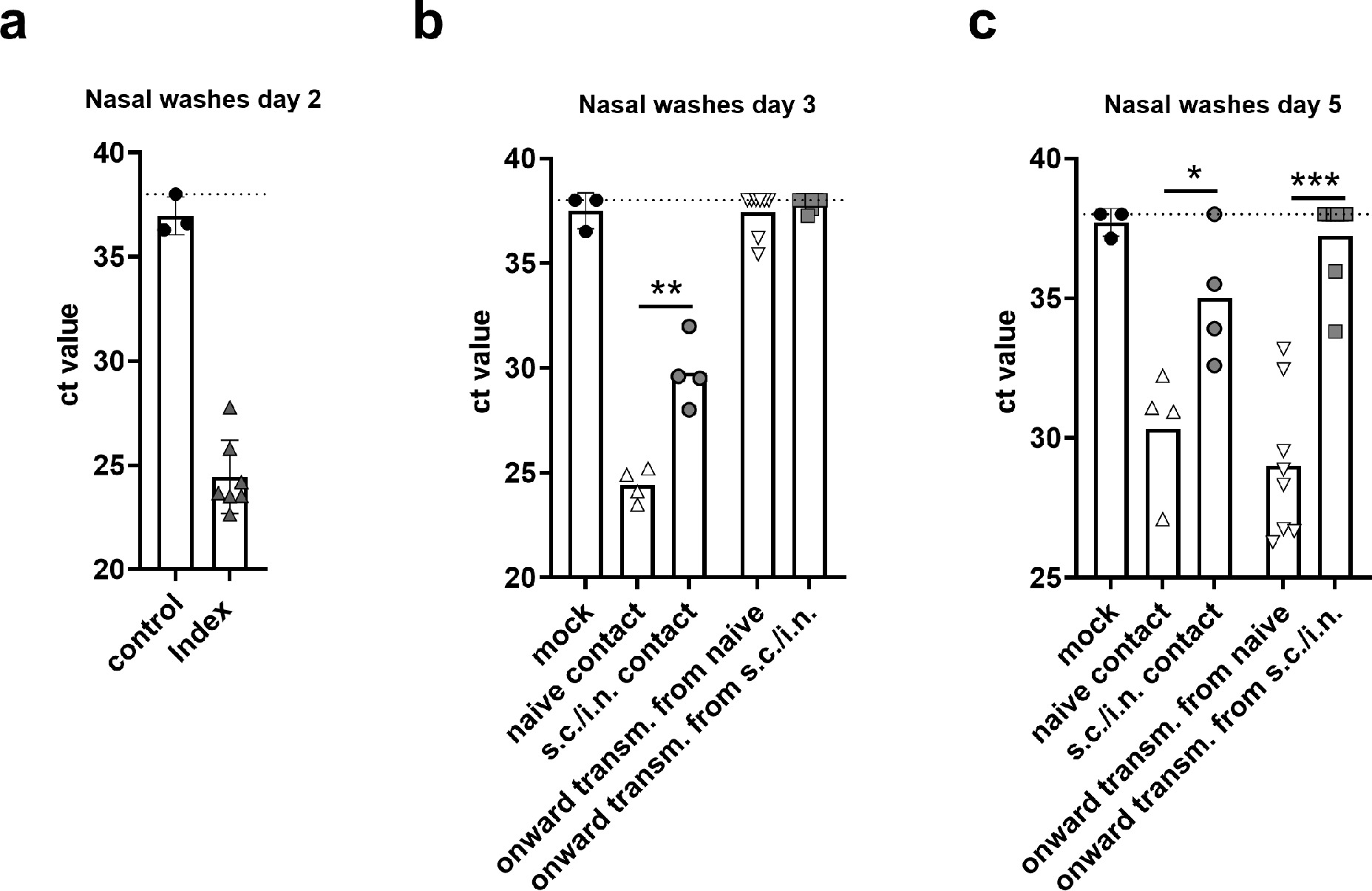

Supplement: Supplementary file 6 [file mmc6.jpg]
